# Supplementary material for: Simulating the effects of mobility restrictions in the spread of SARS-CoV-2 in metropolitan areas in Portugal
Source: PLoS One. 2022 Sep 9;17(9):e0274286. doi: 10.1371/journal.pone.0274286 (PMC9462718; doi:10.1371/journal.pone.0274286)
Supplement: Attachment — Submitted filename: response_to_reviewers.docx [file pone.0274286.s001.docx]

**Manuscript [PONE-D-21-30815]**

**Response to reviewers:**

We thank the reviewers for the comments and suggestions provided. We respond to each point raised below.

**Reviewer #1:**

The manuscript titled "Simulating the effects of mobility restrictions in the spread of SARS-1 CoV-2 in metropolitan 2 areas in Portugal", is well-presented. But there are few suggestions to be considered.

1. there are few grammatical and Syntax errors.

R1: We have revised the manuscript and tried to correct any grammatical and syntax errors. Some changes were made throughout the text, please see revised version of the manuscript.

2. the quality of all figures especially 3 to 6 should be improved.

R2: We understand the point raised and we have revised the figures as requested, improving the image quality. Please see revised figures 3 to 6.

3. Conclusion should be more concise.

R3: Conclusion was revised accordingly, we have removed nonessential text. Please see revised version of the manuscript (page 13).

4. Similarly, abstract should be more focused on the study and its outcome/results. less background information will be enough.

R4: As suggested, the abstract was revised to be more focused and we have removed background information that was not essential in this section. Please see the revised version of the manuscript, abstract section (page 1).

**Reviewer #2:**

In this manuscript the authors simulate the COVID restrictions and their effect on the spread of SARS-CoV-2 in two metropolitan cities Lisbon and Porto of Portugal. The study was based on SEIR model to estimate new daily infections. Four different mobility scenarios ranging from full restrictions to unrestricted full activity, were selected/simulated. The study has practical implications in determining/estimating the future number of infections as well as accepting the restriction practices in masses. The study has a strong hypothesis, the methodology is strong and results are presented in an intelligent fashion.

R5: We thank the reviewer for the positive feedback. The revised version of the manuscript incorporates some changes intended to further improve its quality.
