# Supplementary material for: Simulating the effects of mobility restrictions in the spread of SARS-CoV-2 in metropolitan areas in Portugal
Source: PLoS One. 2022 Sep 9;17(9):e0274286. doi: 10.1371/journal.pone.0274286 (PMC9462718; doi:10.1371/journal.pone.0274286)
Supplement: Attachment — Submitted filename: response_to_reviewers_R2.docx [file pone.0274286.s002.docx]

**Manuscript [PONE-D-21-30815] R2**

**Journal Requirements:**

Please review your reference list to ensure that it is complete and correct.

R0: We have fully revised the reference list and we found no retracted papers. There were changes in the status of 2 publications [6 and 7], which are no longer preprints. References in the list were updated accordingly.

**Response to editor and reviewers:**

We thank the editor and reviewers for the comments and suggestions provided. We respond to each point raised below.

**Additional Editor Comments:**

The manuscript titled "Simulating the effects of mobility restrictions in the spread of SARS-1 CoV-2 in metropolitan 2 areas in Portugal", is well-presented. But there are few suggestions to be considered.

1. there are few grammatical and Syntax errors.

R1: A thorough revision had been done to check for errors and the changes made were marked in the manuscript, please see revised version.

2. the quality of all figures especially 3 to 6 should be improved.

R2: We have improved the quality of the figures, as requested. Please see revised version of the figures 3 to 6.

3. Conclusion should be more concise.

R3: As suggested, conclusion has been revised and made more concise. Please see revised version of the manuscript.

4. Similarly, abstract should be more focused on the study and its outcome/results. less background information will be enough.

R4: As suggested, the abstract was altered and we have removed unessential background information. Please see the revised version of the manuscript.

**Review Comments to the Author**

**Reviewer #3:** The manuscript "Simulating the effects of mobility restrictions in the spread of SARS-CoV-2 in metropolitan areas in Portugal" is well presented. The results are described in good fashion and can be implicated in COVID-19 restriction policies.

Does it need more statistical evaluations along with (SEIR model)?

R5: We thank the reviewer for the overall positive feedback.

Regarding the suggestion for more statistical evaluations along with SEIR model, we would like to enhance that the statistical analysis done had to be adapted to the data available. As mentioned in the manuscript, the number of daily infections are not available at municipal level (Lines 280 and following in the revised version with track changes), therefore no further comparison with the simulations could be done. Another limitation was that the data of people working in essential activities is not connected to the commuting data nor transport type, therefore they had to be used as separate scenarios (Table 1). Considering the characteristics (limitations) of the data and the objectives of the study, we have then decided to apply a time-series clustering analysis method (section 3.2.) which, to our knowledge, had not yet been used in this context, to obtain further understanding of the patterns and trends of the resulting simulations, that could not be obtained otherwise.

**Reviewer #4:** Authors have addressed all the suggested changes, the manuscript can be accepted after fulfillment of Journals requirements.

R6: We thank the reviewer for the positive feedback.
